# Mitotic phosphorylation of SUN1 loosens its connection with the nuclear lamina while the LINC complex remains intact

**DOI:** 10.4161/nucl.36232

**Published:** 2014-08-26

**Authors:** Jennifer T Patel, Andrew Bottrill, Suzanna L Prosser, Sangeetha Jayaraman, Kees Straatman, Andrew M Fry, Sue Shackleton

**Affiliations:** 1 Department of Biochemistry; University of Leicester; Leicester, UK; 2 Core Biotechnology Services; University of Leicester; Leicester, UK

**Keywords:** LINC complex, SUN proteins, SUN1, mitotic phosphorylation, nuclear envelope breakdown

## Abstract

At the onset mitosis in higher eukaryotes, the nuclear envelope (NE) undergoes dramatic deconstruction to allow separation of duplicated chromosomes. Studies have shown that during this process of nuclear envelope breakdown (NEBD), the extensive protein networks of the nuclear lamina are disassembled through phosphorylation of lamins and several inner nuclear membrane (INM) proteins. The LINC complex, composed of SUN and nesprin proteins, is involved in multiple interactions at the NE and plays vital roles in nuclear and cellular mechanics by connecting the nucleus to the cytoskeleton. Here, we show that SUN1, located in the INM, undergoes mitosis-specific phosphorylation on at least 3 sites within its nucleoplasmic N-terminus. We further identify Cdk1 as the kinase responsible for serine 48 and 333 phosphorylation, while serine 138 is phosphorylated by Plk1. In mitotic cells, SUN1 loses its interaction with N-terminal domain binding partners lamin A/C, emerin, and short nesprin-2 isoforms. Furthermore, a triple phosphomimetic SUN1 mutant displays increased solubility and reduced retention at the NE. In contrast, the central LINC complex interaction between the SUN1 C-terminus and the KASH domain of nesprin-2 is maintained during mitosis. Together, these data support a model whereby mitotic phosphorylation of SUN1 disrupts interactions with nucleoplasmic binding partners, promoting disassembly of the nuclear lamina and, potentially, its chromatin interactions. At the same time, our data add to an emerging picture that the core LINC complex plays an active role in NEBD.

## Introduction

The nuclear envelope (NE) is a double membrane that separates the chromatin from the rest of the cell, thereby allowing control of nuclear events such as DNA replication, gene expression and mitotic entry. The outer nuclear membrane (ONM) is contiguous with, and biochemically very similar to, the endoplasmic reticulum (ER). It is further connected to the inner nuclear membrane (INM) at the periphery of nuclear pore complexes (NPCs), the latter apparently restricting the passage of proteins from the ONM to the INM (reviewed in Antonin et al.^1^). As a result, the INM contains a unique set of integral membrane proteins that are anchored through interactions with the nuclear lamina, a fibrous meshwork of A- and B-type lamin intermediate filament proteins that underlies the INM. The nuclear lamina provides structural support to the NE, maintaining nuclear integrity and, together with its associated INM proteins, also provides attachment sites for chromatin.^2^^,^^3^

At the onset of mitosis, the NE is broken down to allow segregation of the duplicated chromosomes through attachment to the mitotic spindle. Nuclear envelope breakdown (NEBD) is induced by partial disassembly of NPCs and the nuclear lamina in prophase.^4^^-^^6^ NPC disassembly leads to loss of the NE permeability barrier and entry of cytosolic proteins into the nucleus, while depolymerization of the lamina weakens the NE, such that it can be physically torn apart by the pulling force of microtubules attached to the NE via dynein.^7^^,^^8^ This process of disassembly is initiated by phosphorylation of components of the NPC and nuclear lamina by mitotic kinases, in particular Cdk1, but also Plk1, Aurora, and NIMA-related kinases. For example, phosphorylation of the nucleoporin Nup98 by multiple kinases, including Cdk1, Plk1, and NIMA kinases, is thought to be an important step in initiating NPC disassembly.^9^ In contrast, Cdk1 is the only mitotic kinase so far shown to phosphorylate nuclear lamina components, including both A- and B-type lamins and the INM proteins lamina-associated polypeptides 1 and 2 (LAP1 and LAP2) and the lamin B receptor (LBR).^10^^-^^12^ These phosphorylation events depolymerize the lamina meshwork,^12^^,^^13^ disrupting its attachment to the INM and to chromatin,^11^^,^^14^ the latter aiding chromatin condensation.

Since the initial phosphorylation studies of nuclear lamina components were performed, the number of known INM proteins has increased dramatically^15^ and protein networks at the INM have been shown to be far more complex than originally anticipated.^3^ One such mammalian INM protein is SUN1, which, along with its paralogue, SUN2, interacts with nesprins, located in the ONM, to form the LINC complex.^16^^-^^18^ The SUN proteins possess a highly conserved C-terminal SUN domain, named due to its homology with Sad1 and UNC-84 proteins from *S. pombe* and *C. elegans*, respectively. The SUN domain resides in the NE lumen where it interacts with the short C-terminal KASH domain of nesprins.

The LINC complex directly connects the nuclear lamina to the cytoskeleton and is vital for a range of functions including cellular mechanics, nuclear positioning and migration (reviewed in^19^). This is achieved through binding of the N-terminal nucleoplasmic domains of the SUN proteins to the nuclear lamina,^16^^,^^17^ while the cytoplasmic domains of giant isoforms of nesprin-1 and nesprin-2 bind to actin via an N-terminal calponin homology domain^20^^,^^21^ or to microtubules and their motor proteins.^22^^-^^24^ In contrast, nesprin-3 and nesprin-4 connect the NE to the cytoplasmic intermediate filament and microtubule networks, respectively.^25^^,^^26^

At the INM, mammalian SUN proteins form multiple interactions, not only with nuclear lamins, but also with other INM proteins including emerin and short nesprin isoforms, via their nucleoplasmic amino termini.^27^ Furthermore, there is evidence that SUN proteins associate with chromatin.^28^^-^^31^ Given the complex network of interactions involving SUN proteins at the INM, we predicted that they may be subject to mitotic phosphorylation in order to disassemble this network and promote NEBD.

In this study, we focused on SUN1 and found that the protein is indeed phosphorylated at the onset of mitosis by both Cdk1 and Plk1 and that mitotic SUN1 loses interaction with A-type lamins, emerin, and nucleoplasmic nesprin isoforms. In contrast, the core LINC complex, involving the SUN-KASH domain interaction, remains intact. Furthermore, a triple phosphomimetic SUN1 mutant has increased solubility and reduced association with the NE. Thus, mitotic phosphorylation of SUN1 plays a key role in regulating SUN1 interactions with the nuclear lamina and is likely to contribute efficient nuclear envelope breakdown.

## Results

### SUN1 is phosphorylated during mitosis

To gain preliminary evidence of SUN1 phosphorylation during mitosis, we compared the electrophoretic mobility of both endogenous and exogenously expressed myc-tagged SUN1 in asynchronous, S phase-arrested or mitotically-arrested HeLa cells. For both endogenous and exogenously expressed SUN1, a slower migrating band was observed, suggestive of phosphorylation, which was only present in mitotic cells (Fig. 1A-C). To confirm that this band-shift represented a phosphorylated form of SUN1, mitotically arrested HeLa cell extracts were treated with λ phosphatase. The band-shift was abolished in treated samples, indicating that the shift was indeed due to phosphorylation of SUN1 (Fig. 1D). Furthermore, we performed a time-course of release from a G2 arrest, induced using the Cdk1 inhibitor RO-3306, and found that the intensity of the band-shift peaked around 30 min to 1 h after release but was almost completely lost by 2 h after release, consistent with the timing of entry and exit from mitosis, as visualized by expression of cyclin A (Fig. 1E). Thus our data indicate that SUN1 is specifically phosphorylated upon entry into mitosis, remains phosphorylated during mitosis and is dephosphorylated upon re-entry into interphase.

**Figure:**
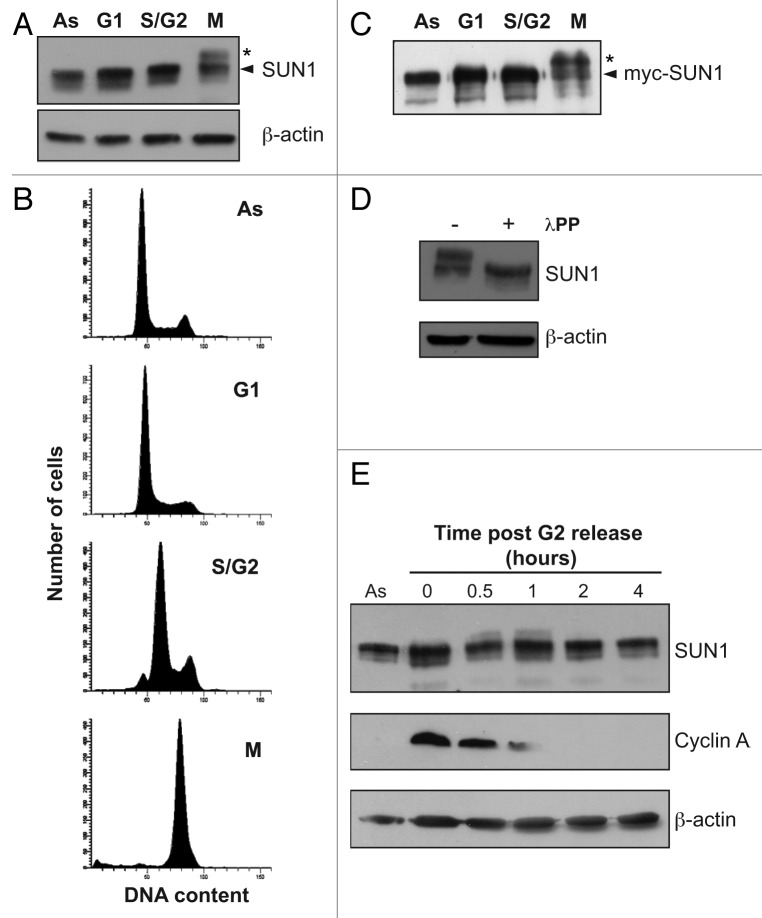
**Figure 1.** SUN1 is phosphorylated during mitosis. (**A-C**) HeLa cells, either untransfected (**A, B**) or transiently transfected with myc-SUN1 (**C**), were arrested in S phase with aphidicolin (S), released from this block for 4 h (S/G2) and arrested in mitosis with nocodazole (M). Cell extracts were immunoblotted along with extracts from asynchronously growing cells (As) and probed with anti-SUN1 (**A**) or anti-myc (**C**) antibodies. Band-shifts are indicated by asterisks. Cells were also analyzed by flow cytometry and the relative proportions in G1, S, and G2/M are indicated (**B**). (**D**) Mitotic HeLa cell extracts, prepared as in (**A-C**), were incubated with λ-phosphatase (λPP) and subjected to immunoblotting with anti-SUN1 antibodies. Probing for cyclin B confirmed that the cells were blocked in mitosis. (**E**) HeLa cells were pre-arrested in S phase using aphidicolin, then blocked in late G2 using Cdk1 inhibitor, RO-3306, and released for up to 4 h, as indicated. Extracts were immunoblotted with anti-SUN1 antibodies. Probing for cyclin A identified mitotic samples.

### SUN1 is phosphorylated at multiple sites within its nucleoplasmic N-terminal domain

To identify mitotic phosphorylation sites on SUN1, we purified GFP-SUN1 from transiently transfected, mitotically arrested HeLa cells by GFP-Trap and performed liquid chromatography-tandem mass spectrometry (LC-MS/MS) on both tryptic and AspN-digested peptides. Combined sequence coverage was 87%, with 86% coverage in the N-terminal nucleoplasmic domain (residues 1–362), which is the region exposed to mitotic kinases in the nucleoplasm. A total of 3 mitotic phosphorylation sites were identified, at S48, S138, and S333, all located within the N-terminal domain of the protein (Fig. 2A). In contrast, no phosphorylation sites were identified in samples from non-synchronised cells, suggesting that the 3 phosphoserines are all mitosis-specific. Furthermore, no phosphorylation of the luminal C-terminal domain was observed in either asynchronous or mitotic samples.

**Figure:**
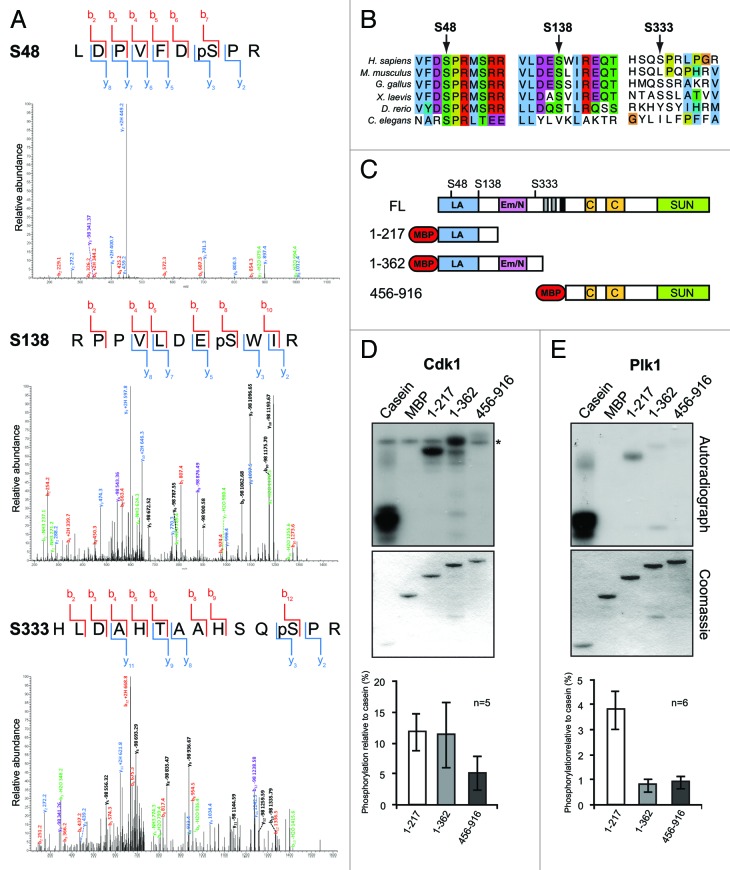
**Figure 2.** SUN1 is phosphorylated at 3 sites by Cdk1 and Plk1. (**A**) GFP-SUN1 was purified by GFP-Trap from transiently transfected HeLa cells and subjected to LC-MS/MS after digestion with trypsin or Asp-N and phosphopeptide enrichment. LC-MS/MS traces for the 3 phospho-serine-containing peptides are shown, along with positions of the detected b-ions and y-ions. (**B**) Sequence alignments of SUN1 from multiple vertebrate species and UNC-84 from *C. elegans*. The phospho-serine residues identified by MS in human SUN1 are indicated. (**C**) Schematic diagram of full-length (FL) SUN1 and the MBP-SUN1 fusions used in in vitro kinase assays. Mapped lamin A (LA) and emerin/nesprin-2 (Em/N2) binding sites are indicated, as are the locations of the identified phosphoserines. Black bar indicates transmembrane domain; gray bars indicate hydrophobic regions. C – coiled-coil domain; SUN – conserved SUN domain. (**D, E**) Purified casein, MBP and MBP-SUN1 fusion proteins were subjected to in vitro phosphorylation by either Cdk1/cyclin B (**D**) or Plk1 (**E**) in the presence of [γ-^32^P]-ATP. Following gel electrophoresis and Coomassie staining, phosphorylated proteins were detected by autoradiography. Asterisk indicates autophosphorylated Cdk1. The proportion of radioactivity incorporated into each sample, relative to the casein control, is presented below. Error bars represent s.e.m.

Comparison of the sequences surrounding each of the phosphoserines with consensus phosphorylation sites of mitotic kinases revealed S48 and S333 as potential Cdk1 phosphorylation sites (consensus S/T*-P-x-K/R) and S138 as a potential Plk1 phosphorylation site (consensus D/E-x-S/T*-Φ) (Fig. 2B). The sequences surrounding both S48 and S138 are highly conserved in vertebrates, with S48 also being found in the *C. elegans* ortholog, UNC-84, supporting their functional importance. In contrast, S333 is poorly conserved and so its significance is less obvious.

To test whether Cdk1 and Plk1 are capable of phosphorylating SUN1, we performed in vitro kinase assays using purified MBP-tagged SUN1 protein fragments. We found that both Cdk1 and Plk1 phosphorylated a SUN1 fragment encompassing residues 1–217, but not residues 456–916, consistent with the presence of phosphorylation sites for both kinases in this N-terminal fragment (Fig. 2C-E). A fragment encompassing residues 1–362 was less well phosphorylated by Plk1, perhaps because this fragment is not correctly folded, causing masking of the phosphorylation sites. Importantly, however, LC-MS/MS analysis of non-radiolabelled versions of both kinase-treated purified N-terminal SUN1 fragments confirmed phosphorylation of the same 3 sites previously identified in vivo and, furthermore, demonstrated that Cdk1 was capable of phosphorylation of S48 and S333, while Plk1 phosphorylated S138 (data not shown).

### Cdk1 and Plk1 phosphorylate SUN1 in vivo

To test the importance of Cdk1 and Plk1 for SUN1 phosphorylation in vivo, we treated mitotically-arrested HeLa cells with Cdk1 or Plk1 inhibitors and assessed whether the band-shift of endogenous SUN1 previously observed in mitotic extracts was lost. As shown in Figure 3A, the Cdk1 inhibitor roscovitine caused a significant reduction in the band-shift and this reduction was enhanced when combined inhibition of Cdk1 and Plk1 was performed. No reduction in the band-shift was observed in the presence of either Aurora A or MEK inhibitors. These data indicate that Cdk1 and Plk1 make a significant contribution to mitotic phosphorylation of SUN1. Of note, migration of SUN1 on the gel did not return to the position observed in asynchronous cells, indicating that additional kinases may also play a role in SUN1 mitotic phosphorylation, or that it may undergo other forms of covalent modification.

**Figure:**
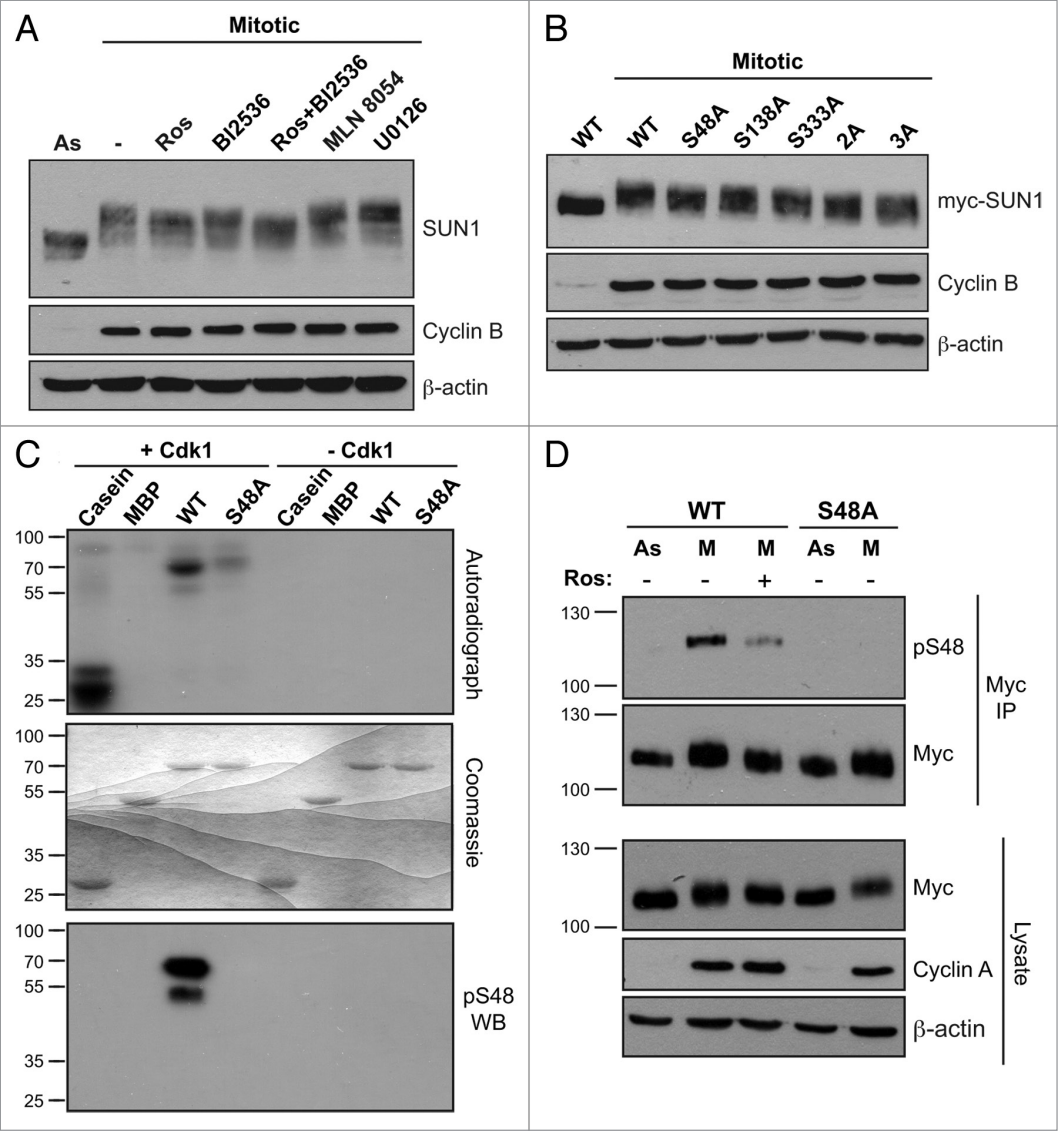
**Figure 3.** SUN1 is phosphorylated by Cdk1 and Plk1 in vivo. (**A**) HeLa cells were pre-arrested in S phase with aphidicolin, released and then arrested in mitosis with nocodazole overnight. The indicated kinase inhibitors were added for the final 4 h of incubation and cell extracts were then subjected to immunoblotting alongside untreated asynchronous (As) samples using the indicated antibodies. Ros – roscovitine. (**B**) HeLa cells were transfected with the indicated wild-type (WT) or mutant myc-SUN1 constructs and extracts from either asynchronous or mitotic cells were prepared and immunoblotted as in A. (**C**) Purified casein, MBP, MBP-SUN1 (residues 1–217) and MBP-SUN1-S48A were subjected to in vitro phosphorylation by Cdk1 in the presence of [^32^P]-γ-ATP. Samples were resolved by SDS-PAGE and dried gels analyzed by autoradiography to identify phosphorylated proteins. Parallel samples were prepared as above, except with the omission of radiolabelled ATP. Samples were immunoblotted using the polyclonal pS48-SUN1 antibody. The antibody detected only phosphorylated wild-type SUN1. (**D**) Cells were transfected with either WT or S48A SUN1 mutant and grown asynchronously (As) or arrested in mitosis (M) as described in A. Some samples were incubated with roscovitine (Ros) for the final 4 h, as indicated. Cell extracts were subjected to immunoprecipitation with myc antibodies and samples immunoblotted with myc or SUN1 pS48 antibodies. Initial lysates were probed with cyclin A antibodies to confirm cell cycle status. Molecular weights (kDa) are indicated.

To investigate the role of the 3 identified phosphoserines in SUN1 phosphorylation, we generated myc-tagged phospho-null mutants of SUN1 by alanine substitution, creating S48A, S138A, and S333A mutants. In addition, we generated a double mutant of the Cdk1 phosphoserines S48 and S333, termed SUN1–2A, and a triple mutant of all 3 serines, termed SUN1–3A. A partial reduction in the band-shift was observed for SUN1-S48A while a more complete loss of the bandshift was seen with the 2A and 3A mutants (Fig. 3B). These data provide further evidence for phosphorylation of serines 48, 138, and 333 in vivo.

We generated a polyclonal phospho-antibody against one of the two highly conserved phosphorylation sites, phospho-S48 (pS48), in order to confirm the phosphorylation site in vivo. We initially verified that the antibody was able to detect bacterially expressed wild-type SUN1, but not an S48A mutant, following incubation with Cdk1 in vitro (Fig. 3C). The antibody was then used to detect exogenously expressed myc-SUN1 following immunoprecipitation from HeLa cells using myc antibodies. The antibody detected myc-SUN1 immunoprecipitated from mitotically arrested HeLa cells, but not from asynchronous cells (Fig. 3D). Furthermore, the signal was significantly reduced following inhibition of Cdk1 activity using roscovitine and recognition of the protein was completely abolished by the S48A mutation. Thus, phosphorylation of S48 by Cdk1 is confirmed in vivo.

### Mitotic SUN1 loses interaction with lamin A/C and emerin, but the LINC complex remains intact

SUN1 participates in multiple interactions at the NE, most of which occur via its nucleoplasmic N-terminal domain at the INM. We therefore sought to determine whether SUN1 interactions with its INM binding partners are disrupted in mitotic cells. SUN1 was immunoprecipitated from either asynchronous or mitotic HeLa cells and probing of immunoprecipitates revealed that interaction with lamin A/C and emerin was disrupted in mitotic cells (Fig. 4A).

**Figure:**
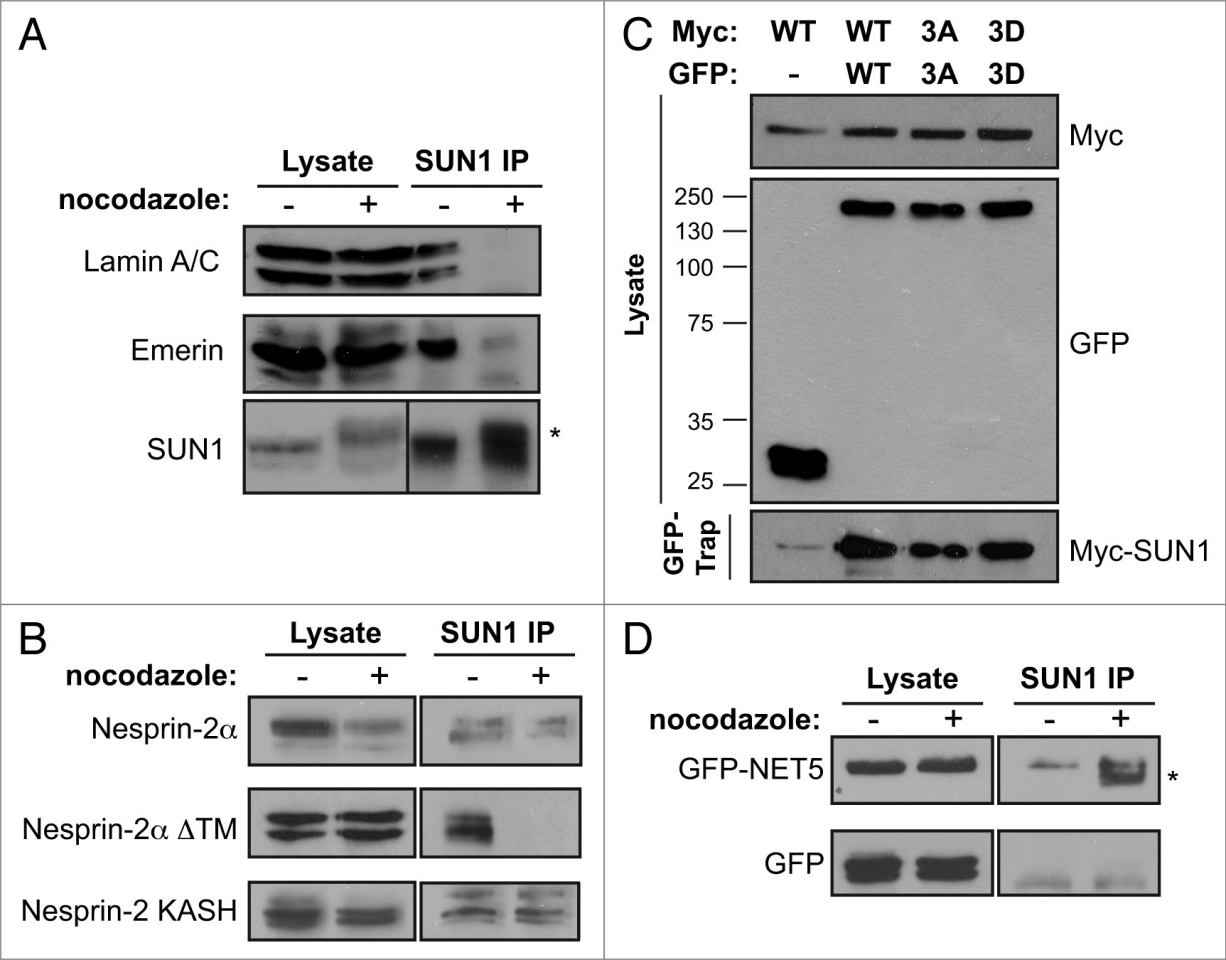
**Figure 4.** SUN1 interactions with lamin A/C and emerin are disrupted in mitosis. (**A**) HeLa cells were either untreated (-) or arrested in mitosis with nocodazole (+). Soluble lysates were then subjected to immunoprecipitation using 2383 anti-SUN1 antibodies. Lysates and immunoprecipitates (IP) were analyzed by immunoblotting using the antibodies indicated. Asterisk indicates the SUN1 bandshift observed in mitotic cell lysates. (**B**) HeLa cells were transiently transfected with GFP-tagged nesprin-2 constructs, as indicated, and immunoprecipitations performed as in A. Samples were immunoblotted using anti-GFP antibodies. (**C**) HeLa cells were transiently transfected with constructs encoding GFP (-), GFP-SUN1 WT, 3A, or 3D, together with the equivalent myc-SUN1 constructs, as indicated. Soluble lysates were subjected to GFP-Trap and co-purified myc-SUN1 proteins detected by immunoblotting. Molecular weights (kDa) are indicated. (**D**) HeLa cells were transiently transfected with GFP or GFP-NET5 and immunoprecipitations performed as in A. Samples were immunoblotted using anti-GFP antibodies. Asterisk indicates a modified form of GFP-NET5 observed in mitotic immunoprecipitates only.

To investigate changes in SUN1 interaction with nesprins, cells were transiently transfected with GFP-nesprin-2α, a short nesprin isoform that is thought to reside partly at the INM, prior to synchronization and immunoprecipitation of SUN1. Nesprin-2α was co-precipitated with SUN1 in both asynchronous and synchronized cells (Fig. 4B). Since SUN1 and nesprin-2α have previously been shown to interact via both their N-termini and C-termini,^27^ we wondered whether both of these interactions were maintained in mitosis. To investigate this and to distinguish between the two interaction sites, cells were transfected with GFP-nesprin-2 KASH, comprising only the lumenal KASH domain that interacts with the C-terminal domain of SUN1, or with GFP-nesprin-2 ΔTM, comprising the nucleoplasmic domain that interacts with the N-terminus of SUN1. Strikingly, we found that SUN1 interaction with the KASH domain of nesprin-2α was maintained, while interaction with the nucleoplasmic domain was abolished in mitotic cells (Fig. 4B).

These data suggested that the LINC complex remains intact during mitosis and we investigated this further by examining SUN1 self-interaction. SUN1 has been reported to oligomerize via the coiled-coil domain located in the luminal C-terminal region^32^ and oligomerization of the N-terminal domain has also been reported.^17^ To test SUN1 self-interaction, we generated a triple phosphomimetic SUN1 mutant, SUN1–3D, by mutating the 3 phosphorylated serines to aspartate residues. Cells were double-transfected with both myc-tagged and GFP-tagged constructs encoding either WT SUN1 or the 3A or 3D mutants, and their interaction observed by GFP-Trap. Both the triple phosphomimetic and phospho-null mutants maintained self-interaction comparable to that of wild-type SUN1 (Fig. 4C), suggesting that the LINC complex does not disassemble during mitosis.

We next wished to determine whether the triple phosphomimetic SUN1 mutant was capable of recapitulating in asynchronous cells the loss of interaction with lamin A/C and emerin previously observed with endogenous SUN1 in mitotic cells. Cells were transfected with GFP-tagged constructs encoding WT SUN1, SUN1–3A, or SUN1–3D and asynchronous cultures subjected to GFP-Trap. Emerin and lamin A/C were co-precipitated equally well by all 3 forms of SUN1, suggesting that phosphorylation of emerin and lamin A/C may also be required for efficient disruption of their interaction with SUN1 (Fig. S1).

### A novel interaction between SUN1 and Samp1/NET5 is maintained during mitosis

Samp1 was recently identified as a human protein that interacts with SUN2.^33^ A short isoform of the protein, that encodes the N-terminal half of the full-length protein, is functionally associated with the LINC complex and localizes to the mitotic spindle.^34^^,^^35^ We were therefore interested to investigate potential interactions with SUN1. For this purpose we transiently expressed a GFP-tagged construct encoding NET5, the rat homolog of Samp1, in HeLa cells and observed its interaction with SUN1 following immunoprecipitation with SUN1 antibodies. GFP-NET5, but not GFP alone, co-precipitated with SUN1 in asynchronous cell cultures (Fig. 4D and data not shown). We also examined the interaction of SUN1 with GFP-NET5 in nocodazole-arrested cells and found that, analogous to the nesprin-2 KASH domain, this interaction was not disrupted in mitotic cells. Interestingly, an additional band, of lower molecular weight, was observed for GFP-NET5 in mitotic cells, suggesting that this protein may also undergo mitosis-specific modification.

### Serine 48 contributes to SUN1 retention at the NE

To further address the role of the identified SUN1 phosphorylation sites in mitotic events, we examined the subcellular localization of myc-SUN1 single, double, and triple phosphomimetic mutants in interphase U2OS cells and compared this with the equivalent phospho-null mutants. Whereas wild-type SUN1 and all phospho-null mutants localized predominantly at the NE, SUN1-S48D was frequently distributed also within the cytoplasm (Fig. 5A). In contrast, there was no obvious mislocalization of SUN1-S138D or SUN1-S333D. Mislocalization of both double and triple mutants, SUN1–2D and SUN1–3D, to the cytoplasm appeared stronger than that of SUN1-S48D (Fig. 5B). To confirm these observations and, to ensure that cytoplasmic localization was not simply a reflection of the level of overall protein expression, we quantified the ratio of nuclear vs. total fluorescence intensity, which confirmed that the S48D, 2D, and 3D mutants had significantly lower levels of relative nuclear staining compared with the other proteins (Fig. 5C). These results suggest that phosphorylation of SUN1, particularly at S48, leads to reduced anchorage of the protein at the NE and its accumulation in the ER.

**Figure:**
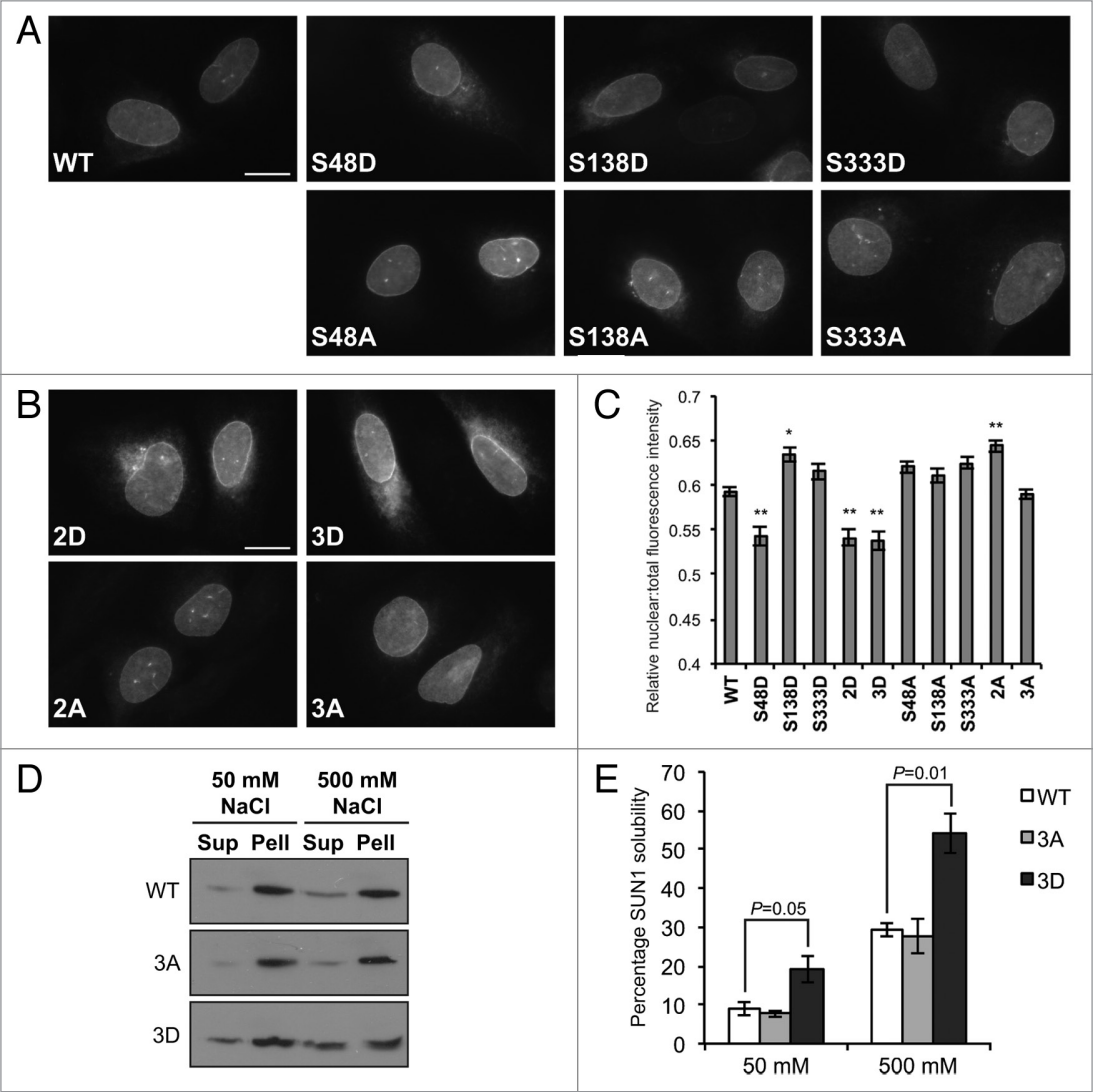
**Figure 5.** Phosphomimetic SUN1 mutants have reduced association with the nuclear envelope. (**A**) U2OS cells grown on coverslips were transfected with the indicated wild-type (WT) or single myc-SUN1 phospho-site mutant constructs for 20 h, fixed in methanol and stained with anti-myc antibodies. Representative images are shown. Scale bar, 10 µm. (**B**) Cells were transfected with double and triple phospho-site mutant constructs and processed as in A. (**C**) Quantification of the relative nuclear vs. total fluorescence intensity for the mutants shown in A and B. (n ≥ 2500 from 2 independent experiments; +/− s.e.m.). * *P* value < 10^−4^, ** *P* value < 10^−5^ when compared with WT. (**D**) HeLa cells were transiently transfected with wild-type SUN1 or triple phospho-null (3A) or phosphomimetic (3D) mutants and proteins were extracted using either 50 mM or 500 mM salt buffer. Following centrifugation, supernatants (Sup) and pellets (Pell) were immunoblotted and probed with myc antibodies to determine protein solubility. (**E**) Densitometric analysis of panel D, showing the percentage of protein in the supernatant, representing the soluble fraction (n = 3; +/− s.e.m.). Significant *P* values, calculated by Student’s *t* test are indicated.

To extend these findings, we assessed the solubility of the SUN1 triple phospho-null and phosphomimetic mutants, SUN1–3A and SUN1–3D, by extraction in low and high salt Triton buffers. While SUN1–3A displayed similar solubility to that of wild-type SUN1, the solubility of SUN1–3D was increased 2-fold in both low and high salt buffers (Fig. 5D-E). Thus, we propose that SUN1 phosphorylation leads to a reduced ability to bind to factors that anchor the protein at the NE during interphase.

## Discussion

In this study, we have demonstrated that SUN1 undergoes mitosis-specific phosphorylation on at least 3 sites. Furthermore, we have identified S48 and S333 as targets of Cdk1, whist S138 is a target of Plk1 phosphorylation. In agreement with our findings, S138 and S333 have also been identified as sites of mitotic phosphorylation in several phosphoproteomics studies.^36^^,^^37^

### SUN1 phosphorylation weakens its association with the nuclear lamina

All 3 identified phosphosites lie within the N-terminal domain of SUN1, which is located in the nucleoplasm and therefore exposed to mitotic kinases after their entry into the nucleus during prophase. In contrast, no phosphosites were identified in the C-terminal domain, which is located in the NE lumen and therefore not accessible to these kinases. The nucleoplasmic domain of SUN1 has binding sites for several nuclear lamina and INM components, including lamin A/C, emerin, and short nesprin isoforms,^27^ which could be regulated by phosphorylation. In support of this, we found that SUN1 interaction with all of these proteins was lost during mitosis, indicating that phosphorylation of SUN1 is likely to contribute to disassembly of the protein networks that play a vital role in organization and integrity of the interphase nucleus. We were unable to recapitulate these findings using a triple phosphomimetic SUN1 mutant; however, there are several potential explanations for this. First, loss of interaction may require phosphorylation of multiple components, including lamin A/C which is known to be phosphorylated during mitosis,^12^ and these experiments were performed using asynchronous cells, where all proteins were predominantly non-phosphorylated. Second, since SUN proteins exist in a trimeric state,^38^^,^^39^ the presence of endogenous SUN1 could mask the effects of the exogenously expressed mutants through the formation of heterotrimers. On the other hand, previous studies have shown that overexpressed exogenous SUN1 is capable of displacing endogenous SUN proteins from the NE (see ref. 16; Haque and Shackleton, unpublished observations), thus potentially minimizing this effect. Another possibility is that phosphorylation of additional undiscovered sites may be required for efficient loss of interaction. Further sites could lie within the 13% of the N-terminal domain not covered in our MS analysis. Indeed, a putative Aurora A phosphorylation site lies at serine 52, which falls within a region that was not covered in our MS study.

Despite no loss of interactions with the triple phosphomimetic SUN1 mutant, we observed a significant increase in its solubility compared with both wild-type SUN1 and a triple phospho-null mutant, together with a reduced association with the NE. This effect appeared to be largely controlled by phosphorylation of S48 since the S48D single phosphomimetic mutation had a similar effect on SUN1 localization to the NE compared with the triple mutation. Similar changes in other NE proteins have been shown to occur in response to mitotic phosphorylation: phosphorylation of lamins results in their solubilization and accumulation in the nucleoplasm during prophase,^5^^,^^12^^,^^13^ while LBR is released from its anchorage at the INM and redistributes to the ER prior to NEBD.^40^ Thus a role for SUN1 phosphorylation in loosening connections with the nuclear lamina and/or chromatin is likely.

Together, these findings suggest that disruption of the lamin A/C and emerin interaction may not be the major determinant of SUN1 solubilization. Indeed, lamin A/C and emerin are not the key binding partners responsible for anchorage of SUN1 at the NE since SUN1 maintains localization at the NE in cells *Lmna*^−/−^ cells.^17^ In contrast, emerin and nesprin-2 are mislocalized to the ER in these cells.^41^^,^^42^ The major factor(s) that tether SUN1 at the INM have yet to be identified, however, the fact that SUN proteins across a wide range of species interact with chromatin (reviewed in ref. 43) suggests that this may play a significant role in SUN1 anchorage at the INM.

### Roles for the LINC complex during mitosis

In contrast to interactions mediated by the N-terminal domain of SUN1, we found that the central LINC complex SUN-KASH interaction remains intact in mitotic cells, suggesting that it plays a functional role during mitosis. In support of this, a recent study demonstrated that SUN1 and SUN2 are required for the formation of prophase nuclear invaginations (PNEIs) and for the separation of nuclear membranes from chromatin after NEBD.^44^ PNEIs are generated through the pulling forces of microtubules associated with the NE via dynein, resulting in tearing of the NE.^7^^,^^8^ Importantly, their formation was also perturbed by disruption of the LINC complex with a dominant-negative KASH domain mutant, indicating that the LINC complex must remain intact in order to fulfill this function.^44^ Thus, the LINC complex appears to be responsible for microtubule association with nuclear membranes throughout the cell cycle.

We also observed a novel interaction between SUN1 and NET5/Samp1 that was maintained during mitosis. The long isoforms of Samp1 have previously been shown to interact with SUN2 and are likely to contribute to nuclear movement in migrating fibroblasts.^33^ However, a short Samp1 isoform localizes around the spindle poles during mitosis and has been proposed to be a component of a membranous structure that may contribute to spindle structure or function.^34^ The fact that SUN1 maintains interaction with NET5/Samp1 during mitosis suggests that the LINC complex may also be part of this structure. SUN1 has also been reported to be concentrated close to the spindle poles in metaphase, however, this may represent NE membranes that have been recently separated from the chromatin during NEBD.^17^ Moreover, there appeared to be a preferential interaction with a faster-migrating form of NET5 in mitotic SUN1 samples, suggestive of a modified complex that could have unique functions during mitosis.

Thus, our findings indicate multiple roles for SUN1 during mitosis. We propose a model whereby phosphorylation of SUN1 on entry into mitosis disrupts interactions with nucleoplasmic binding partners, thereby promoting nuclear lamina disassembly and chromatin condensation. Loss of these interactions further enables nuclear membranes to be separated from the condensed chromatin after NEBD. On the other hand, maintenance of the LINC complex may promote dynein association with the NE in prophase, which in turn promotes timely NEBD through the action of NE-associated microtubules. This model suggests that the LINC complex, along with the NPC component Nup133,^45^ may provide a prophase anchorage site for dynein.

## Materials and Methods

### Plasmids

The human SUN1 (hSUN1) cDNA, encoding the 916 residue full-length protein, was generated by ligating together cDNA sequences obtained from two different sources. The sequence encoding residues 1–362 was PCR-amplified using IMAGE clone 40148216 as a template and introducing a BglII site at codon 356–357. The region encoding residues 349–916 was generated by RT-PCR amplification from U2OS cell RNA, again incorporating a BglII site at codon 356–357. The BglII site was then used to ligate the two fragments together and the complete cDNA was cloned by recombination into pLEICS-20 (N-terminal myc tag) or pLEICS-21 (N-terminal GFP tag) mammalian expression vectors, using the University of Leicester Protex cloning service. MBP-hSUN1 fusion vectors were generated by PCR-amplification of the relevant regions of the SUN1 cDNA and cloning by recombination into pLEICS-10 (N-terminal MBP tag). Site-directed mutagenesis, to produce phospho-null or phosphomimetic SUN1 mutants, was performed by the Protex cloning service. The pEGFP-nesprin 2α, pEGFP-nesprin 2α ΔTM, and pEGFP-nesprin 2α KASH constructs, provided by C Shanahan, have been described previously (Zhang et al. 2002, and Zhang et al. 2005). The pEGFP-NET construct was kindly provided by E. Schirmer (University of Edinburgh, Edinburgh, Scotland, UK).

### Antibodies

Rabbit anti-human SUN1 (Atlas) and mouse monoclonal β-actin antibodies were obtained from Sigma. Mouse anti-Myc antibody was purchased from Zymed Laboratories Inc Mouse anti-cyclin B1, mouse anti-cyclin A and goat anti-lamin A/C (6215) antibodies were purchased from Santa Cruz Biotechnologies. Rabbit anti-GFP antibody was obtained from Abcam. Rabbit anti-emerin antibody was kindly provided by G. Morris (Center for Inherited Neuromuscular Disease, Oswestry, UK). Anti-human SUN1 2383 has been reported previously.^27^

SUN1 serine 48 (pS48) phospho-antibodies were generated by immunization of rabbits with a peptide corresponding to residues 42–55 of human SUN1, phosphorylated at serine 48. Immunizations and antibody purification, using peptide columns, were performed by Eurogentec.

### Cell culture, transfection, and drug treatments

HeLa and U2OS cells were routinely grown in Dulbecco’s modified Eagle medium supplemented with 10% FBS, 1% Glutamax, and antibiotics (growth medium) at 37 °C and 5% CO_2_. Cells were transiently transfected using Lipofectamine 2000 (Invitrogen), according to the manufacturer’s instructions. For synchronization experiments, cells were first pre-arrested in S phase by incubation for 16 h in growth medium supplemented with 1.6 µg/ml aphidicolin (Sigma). Cells were then washed with PBS and incubated with growth medium for 4 h. To arrest in mitosis, 500 ng/ml nocodazole (Sigma) was then added to the medium and the cells incubated for a further 16 h at 37 °C. Mitotic cells were collected by shake-off. Synchronization was confirmed by flow cytometry. To arrest in G2, 10 µM RO-3306 (Calbiochem) was added to the medium 4 h after release from aphidicolin and cells incubated for up to 4 h at 37 °C. For kinase inhibitor studies, the inhibitors were added to nocodazole-arrested cells 4 h prior to harvesting at the following concentrations: 100 µM roscovitine (Calbiochem), 1 µM MLN 8054 (SelleckChem), 100 nM BI2536 (Axon Medchem) and 10 µM U0126 (Cell Signaling), along with 10 µM MG132 (Calbiochem) to prevent protein degradation.

### Flow cytometry

Cells were permeabilized and fixed in ice-cold 70% ethanol then stored at -20 °C for a minimum of 24 h. Cells were then washed and stained with propidium iodide (Calbiochem) staining solution (propidium iodide 50 µg/ml, RNase A 10 µg/ml, PBS). The cells were transferred to round-bottom tubes and incubated for 1 h at 37 °C. The DNA profile for each sample was obtained by FACS (BD Biosciences FACSCanto II) using FACSDiva 6.0 software (BD Biosciences) for acquisition and analysis.

### Cell extracts

Total cell extracts for immunoblotting were prepared by resuspending trypsinized, pelleted cells in phosphate-buffered saline (PBS), followed by boiling in an equal volume of 2 × Laemmli buffer.

Alternatively, soluble cell lysates were prepared by incubating the scraped and pelleted cells in lysis buffer (10 mM HEPES-KOH pH 7.4, 100 mM NaCl, 5 mM EDTA, 1% Triton X-100, 1 × complete protease inhibitor cocktail (Roche), 2 mM AEBSF (Melford), 5 mM NaF, 50 mM β-glycerophosphate) for 30 min on ice. Samples were sonicated for 3 cycles of 15 s and 2 µm amplitude in an MSE Soniprep 150, then centrifuged at 10 000 g (20 000 g for GFP-Trap) for 10 min at 4 °C. The soluble supernatant fraction was further processed for immunoprecipitation or GFP-Trap.

In some instances, soluble cell extracts were treated with lambda protein phosphatase (Sigma), according to the manufacturer’s instructions, prior to immunoblotting.

To examine solubility of transiently expressed myc-SUN1 mutants, whole cell pellets were resuspended in high or low salt concentration buffers (10 mM Tris pH 7.4, 2 mM MgCl_2_, 1% Triton X-100, 1 × complete protease inhibitor cocktail (Roche), 1 × PMSF; supplemented with either 50 [low] or 500 [high] mM NaCl). Samples were incubated on ice for 15 min and then centrifuged at 10 000 g and 4 °C. The supernatants were transferred to new tubes and boiled in an equal volume of Laemmli buffer. Pellets were resuspended in a double volume of 2 × Laemmli buffer and also boiled.

### GFP-Trap, immunoprecipitation, and immunoblotting

For identification of SUN1 phosphorylation sites by mass spectrometry, large scale GFP-trap was performed using 12 10-cm plates of asynchronous or nocodazole-arrested HeLa cells transiently expressing GFP-SUN1. Soluble lysates were processed by GFP-Trap (Chromotek) as described previously,^46^ resolved on 10% polyacrylamide gels and then stained with Brilliant Blue G-Colloidal concentrate electrophoresis reagent (Sigma), according to the manufacturer’s instructions.

For co-immunoprecipitation studies, soluble lysates were prepared from untransfected HeLa cells, or those transfected with the appropriate GFP-hSUN1, myc-hSUN1, GFP-nesprin, or GFP-NET5 constructs, and immunoprecipitated as described previously (Haque et al. 2006) using 2 µg of hSUN1 2383, myc, or GFP antibodies. 5% of the initial lysate was retained for immunoblot analysis.

SUN1 bandshifts were resolved on 6% polyacrylamide gels, while all other samples were resolved on 7.5% or 10% gels. This was followed by semi-dry transfer onto nitrocellulose membrane. Membranes were probed using the appropriate primary antibodies and dilutions: hSUN1 Atlas (1:500), lamin A/C (1:2000), emerin (1:2000), cyclin B1 (1:200), cyclin A (1:200), pS48 (1:50), myc (1:500), GFP (1:6000), and β-actin (1:50 000). Primary antibodies were detected using horseradish peroxidase-conjugated secondary antibodies (Sigma), and visualized using ECL reagents (Geneflow).

### Immunofluorescence microscopy

U20S cells were grown on coverslips, transfected with the appropriate myc-SUN1 construct and then fixed directly in ice-cold methanol for a minimum period of 10 min at -20 °C. Samples were then analyzed by immunofluorescence microscopy as previously described^27^ using anti-Myc antibodies (1:500). The primary antibodies were detected using donkey anti-mouse AlexaFluor 488 secondary antibody (1:500; Invitrogen) and DNA was stained with 4′,6-diamidino-2-phenylindole (DAPI). Quantification of wild-type, phospho-null and phosphomimetic SUN1 mutant localization was performed using an Olympus Scan^R microscope with a 20 × objective. At least 1000 nuclei, each from 2 independent experiments, were selected at random by their DAPI signal, and the total fluorescence intensity of myc-hSUN1 staining (488 nm) was measured within the DAPI-stained region (nuclei), with a threshold of 75 intensity units to allow capture of only transfected cells. The signal 50 pixels around the edge of the nuclei was also measured, to represent the signal in the cytoplasm. The ratio of nuclear vs. total (nuclear + cytosplasmic) fluorescence intensity was calculated for each sample and statistical significance calculated by Student’s *t* test.

### Mass spectrometry

Proteomics was performed by the University of Leicester Proteomics Facility (PNACL), as described previously.^46^ In-gel trypsin and Asp-N digestion was performed on excised bands of interest. Phosphopeptide enrichment was performed using Immobilized Metal Affinity Chromatography (IMAC). PHOS-Select iron affinity gel (Sigma) was equilibrated with 5 × 1 ml IMAC load/wash buffer: 0.25 mM acetic acid, 30% (v/v) acetonitrile. Samples were concentrated using a speedvac (Thermo Electron Corp), resuspended in 500 μl of IMAC load-wash buffer, and 80 μl of 50% slurry of the resin was added directly to each. Following incubation for 1 h at room temperature, the suspensions were transferred to spin columns (MoBiTec GmbH) and centrifuged for 30 s at 8200 g. The flow-through (unbound peptides) were collected and concentrated on a speedvac centrifuge to small volume for further liquid chromatography-mass spectrometry (LC-MS)/MS analysis. The columns were washed twice with 200 μl of IMAC load/wash buffer and once with 200 μl of HPLC-grade H2O. Phospho-peptides bound to the resin were eluted with 2 × 100 μl solution composed of 0.4 M ammonium hydroxide, 30% (v/v) acetonitrile. The eluate was concentrated on a speedvac to a volume of 20 μl and analyzed by LC-MS-MS analysis. Spectra of putative phosphopeptides were interrogated manually to determine the assignment of the site of modification.

### Recombinant protein purification and in vitro kinase assays

MBP fusion proteins were produced by expression of the appropriate pLEICS10-hSUN1 plasmids in *E. coli* BL21 and purification with amylose resin (New England Biolabs). For radiolabelled kinase assays, equal amounts (1–2 µg) of each MBP fusion protein, bound to amylose resin, or purified casein (Sigma) were incubated with 1 μCi of [γ-^32^P]-ATP in kinase buffer (50 mM Hepes-KOH pH 7.4, 5 mM MgCl_2,_ 5 mM β-glycerophosphate, 5 mM NaF, 4 μM ATP, 1 mM DTT) and 5 μg of purified Cdk1/cyclin B or Plk1 (Merck Millipore) for 30 min at 30 °C. For non-radiolabelled kinase assays destined for MS analysis, [γ-^32^P]-ATP was omitted and reactions were incubated for 4 h at 30 °C. Reactions were stopped by addition of 25 μl of Laemmli buffer and analyzed by SDS-PAGE and autoradiography or further processed for LC-MS/MS analysis.

Protein bands from the dried Coomassie-stained gels were excised and placed in scintillation vials along with OptiPhase HiSafe scintillation fluid (Perkin Elmer). Radioactivity was measured in counts per minute (cpm) on an LS 6500 multipurpose scintillation counter (Beckman Coulter). Percentage phosphorylation for each hSUN1 variant was calculated relative to casein.

## Supplementary Material

Additional material

## Abbreviations:

ER: endoplasmic reticulum
INM: inner nuclear membrane
LINC: Linker of nucleoskeleton and cytoskeleton
MBP: maltose binding protein
MS: mass spectrometry
NE: nuclear envelope
NEBD: nuclear envelope breakdown
NPC: nuclear pore complex
ONM: outer nuclear membrane

## Notes

10.4161/nucl.36232
