# Supplementary material for: Mitotic phosphorylation of SUN1 loosens its connection with the nuclear lamina while the LINC complex remains intact
Source: Nucleus. 2014 Aug 26;5(5):462–73. doi: 10.4161/nucl.36232 (PMC4164488; doi:10.4161/nucl.36232)
Supplement: Additional material [file nucl-5-462-s01.pdf]

Landes Bioscience
www.landesbioscience.com

Supplemental Material to:

Jennifer T Patel, Andrew Bottrill, Suzanna L Prosser, Sangeetha Jayaraman, Kees Straatman, Andrew M Fry, and Sue Shackleton

Mitotic phosphorylation of SUN1 loosens its connection with the nuclear lamina while the LINC complex remains intact

Nucleus 2014; 5(5)
http://dx.doi.org/10.4161/nucl.36236

http://www.landesbioscience.com/journals/nucleus/article/36236/

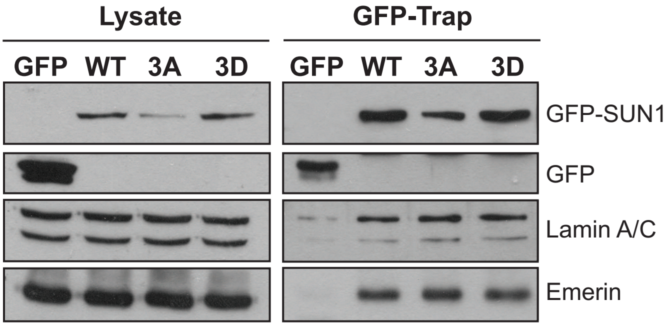

Figure S1. SUN1-3D triple phosphomimetic mutant retains interaction with lamin A/C and emerin in asynchronous cells. Hela cells transiently expressing GFP or GFP-SUN1 WT, 3A or 3D, as indicated, were subjected to GFP-Trap. Samples were immunoblotted, along with initial lysates, to detect the GFP-tagged proteins and co-precipitating binding partners lamin A/C and emerin.
